# Length–weight relationship and condition factor of giant tiger shrimp, *Penaeus monodon* (Fabricius, 1798) from four breeding families

**DOI:** 10.1186/s40064-016-2979-6

**Published:** 2016-08-08

**Authors:** Yundong Li, Falin Zhou, Zhenhua Ma, Jianhua Huang, Shigui Jiang, Qibin Yang, Tao Li, Jian G. Qin

**Affiliations:** 1 South China Sea Resource Exploitation and Protection Collaborative Innovation Center, Sun Yat-Sen University, Guangzhou, 510006 China; 2 South China Sea Fisheries Research Institute, Chinese Academy of Fishery Sciences, Key Laboratory of South China Sea Fishery Resources Exploitation and Utilization, Ministry of Agriculture, Guangzhou, 510300 China; 3 College of Fisheries and Life Science, Shanghai Ocean University, Shanghai, 201306 China; 4 School of Biological Sciences, Flinders University, GPO Box 2100, Adelaide, SA 5001 Australia

**Keywords:** *Penaeus monodon*, Length–weight relationships, Condition factor, Breeding family

## Abstract

**Background:**

Length–weight relationships and condition factors of giant tiger shrimp Penaeus monodon (Fabricius, 1798) from four breeding families (family S: South China seas family, family A: African family, family SA: ♂ South China seas family × ♀ Africa family, family AS: ♂ Africa family × ♀ South China seas family) were evaluated in this study.

**Findings and conclusion:**

Length–weight relationships can be expressed as W = 0.0239BL^2.789^ (*R*^2^ = 0.8977) in family S, W = 0.0206BL^2.9107^ (*R*^2^ = 0.9107) in family A, W = 0.0211BL^2.831^ (*R*^2^ = 0.8869) in family SA, and W = 0.0249BL^2.781^ (*R*^2^ = 0.9159) in family AS. The growth of *P. monodon* from four breeding families follows a negative allometric trend. Fulton’s body condition factor (K) was not significantly different in males, while in females, the highest K (3.07) was observed in family AS, and the lowest K was found in family A (1.88). Results from the present study indicate that the cross group family AS (♂ Africa family × ♀ South China seas family) has obvious heterosis in females. This may suggest that the direction of further breeding of *P. monodon*, should be conducted by using Africa family as male parent, and South China seas family as female parent. Results from the present study will provide valuable information on selective breeding in *P. monodon*. Methodology used in the present study can also be applied in other similar species.

## Background

Length–weight relationships (LWRs) have important implications for fishery management and aquaculture practice (Erzini 1994; Guo et al. 2014). Length–weight regressions have been extensively used to estimate animal weight from length due to the technical constraint in the field (Nie et al. 2014). Fish farmers commonly use fish weight gain to evaluate profit gain and scientists usually adopt length measurements to assess fish growth performance in the field. In selective breeding, LWR is a useful measure for body condition in selected species and to compare morphological differences between populations in different regions (Nie et al. 2013). The LWR has also been applied to compare growth and population parameters (such as birth, immigration, death, emigration) among different shrimp families (Philipose et al. 2013).

Fulton’s condition factor (K) is often used to quantify an animal’s physical wellbeing, and considered to be a useful complement for growth estimate in crustaceans (Rochet 2000). It is also an important parameter for the management of culture systems (Araneda et al. 2008). In fish ecology, the condition index (Bolger and Connolly 1989) is used to monitor the population response to environmental change over time and to assess the overall health, productivity (Richter 2007), lipid content and growth rate (Rister et al. 2000; Stevenson and Woods 2006; Rypel and Richter 2008). The condition factor has also been applied for assessing the overall biotic and abiotic conditions for shrimp growth (Gopalakrishnan et al. 2014).

The giant tiger shrimp *Penaeus monodon* belongs to the family Penaeidae and is widely distributed in the Indo-West Pacific Ocean (Holthuis 1980). The growth of *P. monodon* mainly depends on the sex, developmental stage, and environments (Kumlu et al. 1999; Prasad 2001). The most common body measurements in penaeid shrimp are the carapace length (CL), body length (BL), and body weight. In the present study the LWR and condition factor of *P. monodon* in four breeding families were evaluated, thus the objective of the present study was to provide baseline data on morphological measurement, LWRs and condition factor of *P. monodon* from four families. Results from the present study will provide valuable information on selective breeding in *P. monodon*.

## Methods

### Broodstocks cultivation and mating

The broodstocks used in this study were wild shrimp collected from Mozambique Channel, Africa, and the coastline of Sanya City, People’s Republic of China. Females and males were separately cultured in cement tanks (7 × 3 × 2 m). The environmental parameters were kept at 28–33 °C, 5–9 mg dissolved oxygen L^−1^, 28–35 ‰ salinity and 7.8–8.2 pH The broodstock were fed with a conditioning diet of fresh frozen squid and a clam worm (*Nereis succinea*). Female broodstock was artificially inseminated 2-days post-moult using spermatophores extracted from male broodstocks. Each female was unilaterally eyestalk ablated using heated wire snips. The eye was tagged for individual identification and then returned to the tank immediately after artificial insemination. Each batch of eggs was collected, washed and transferred to a tray and nauplii were hatched in the following morning. Then, the nauplii were separated into family batches.

### Larval rearing and fluorescent marked

The nauplii from different families were separately cultured in four replicated rearing tanks at a density of 40,000 nauplii per tank, feed with diatom and water-soluble compound feed three times a day. The rearing water was filtrated and sterilized by ultraviolet light. Water was daily exchanged and maintained at 20–50 cm deep. The water quality and culture environment in all tanks during this process were same, kept at 28–30 °C, 28–32 ‰ salinity, 7.8–8.0 pH, more than 6 mg dissolved oxygen L^−1^. At 15 days post metamorphosis, offspring of each family was relocated into plankton mesh cages (40 mesh, 3-m length × 3-m width × 2-m deep) suspended in a communal pond (4050 m^3^). At this stage, the environmental parameters were kept constant, the shrimps were fed with larvi-artemia and commercial pellet feed (CP Shrimp Feed, Thailand) three times a day at 00:07, 1200, and 1730 hours. At 16 weeks old, each shrimp was individually tagged at an average weight of 3 g using visible implant fluorescent elastomers. Visible implant elastomer tags (NMTTM, Shaw Island, Washington, USA) were injected intramuscularly into the dorsal, ventral left and ventral right portions of the last abdominal segment in each shrimp. The tag colors were combined in a way that each family could be easily distinguished (Krishna et al. 2011). All experiment was carried out in a communal grow-out cement pond (30-m length × 30-m width × 4.5-m deep) to ensure that the rearing environment and condition were identical. The environment was kept at 25–32 °C, 25–35 ‰ salinity, 7.8–8.2 pH, 6–8.5 mg dissolved oxygen L^−1^. Cleaning the pond was performed by discharging the bottom 10–30 cm of water every day. The feeds were commercial pellet feed and the animals and plants in the pond. Dissolved oxygen and water temperatures were measured by a portable water quality meter (HACH, USA), while salinity and pH were measured using a hand refractometer (ATAGO, Japan), and a portable pH meter (SANXIN, Shanghai), respectively.

### Data measurement

According to the farm records, the study period was from April 10 to October 13, 2014. The water quality recorded at the sampling sites was within the optimum range for *P. monodon* growth. Shrimp were sampled and measured after hatching 180 days. In this study, a total of 1120 specimens belonging to four groups were measured. These groups were: group S composed of 412 individuals from South China Sea parents, group A composed of 358 individuals from Mozambique channel (Africa) parents, group SA composed of 185 individuals from South China Sea male and Mozambique channel female parents and group AS composed of 165 individuals from Mozambique channel male and South China Sea female parents. The LWR was estimated for body wet weight (W) and BL. The CL, carapace width and carapace height (CH) of *P. monodon* were measured and analyzed. All the length parameters were measured with a ruler to the nearest 0.1 cm. Wet weights were measured with an electronic balance to the nearest 0.1 g. Sex was determined by checking the external genital organs.

### Statistical analysis

The relationships between BL and wet weight (W) were calculated by the power regression W = a × BL^b^. The association degree between BL and W was calculated by the determination coefficient (r^2^). One sample *t* test was used to compare the *b* value in this study. Fulton’s condition factor (K) was estimated from the individual length and individual weight in the sample estimated from this equation, K = 100 W/BL^b^, where K = condition factor, W = mean weight (g), BL = body length (cm), and b value was derived from the W = a × BL^b^. Variables presented in this study were expressed as mean ± SD, and one-way ANOVA was used to compare the differences of CL, CW, CH, and Fulton’s condition factor between four shrimp families (PASW Statistics 20.0). When a significant family effect was found, Tukey’s test was performed for multiple range comparisons (*P* < 0.05).

## Results and discussion

bl and weight relationships calculated for 4 shrimp groups are shown in Table 1. The LWRs of *P. monodon* sampled from four breeding groups follows a negative allometric trend. In addition, the b value for males of family A (b = 2.9107) was Significant difference with other families and the females of family SA (b = 2.9450) was also with the differences in b-values compared with others groups.

**Table 1:** Descriptive statistics and estimated parameters of length–weight relationships for *P. monodon* from four breeding families

Family/sex	n	Body length (cm)		Wet weight (g)		a	b	r^2^
		Min.	Max.	Min.	Max.			
South								
Male	210	6.1	12.0	2.87	27.69	0.0230	2.8031	0.9011
Female	202	6.1	12.6	2.96	29.15	0.0256	2.7640	0.8917
Pooled	412	6.1	12.6	2.96	29.15	0.0239	2.7890	0.8977
Africa								
Male	190	4.2	11.9	1.08	25.60	0.0186	2.9107	0.9107
Female	168	4.4	12.2	1.57	26.52	0.0234	2.7950	0.9009
Pooled	358	4.2	12.2	1.08	26.52	0.0506	2.8510	0.9107
South-Africa								
Male	92	4.0	11.8	1.48	24.16	0.0266	2.7280	0.8989
Female	93	6.3	11.4	3.25	25.79	0.0163	2.9460	0.8636
Pooled	185	4.0	11.8	1.48	25.79	0.0211	2.8310	0.8869
Africa-South								
Male	80	6.3	11.7	3.40	22.85	0.0297	2.7050	0.8945
Female	85	6.6	11.6	4.28	24.50	0.0204	2.8687	0.9388
Pooled	165	6.3	11.7	3.40	24.50	0.0249	2.7810	0.9159

n: Number of shrimp, max: maximum length, min: minimum length, a: constant of the relationship, b: slope of the relationship, r^2^: coeffcient of determination

The CLs of male and female shrimp in family S were significantly greater than in other three families (*P* < 0.05, Table 2). The largest CW was found in family S both in males and females. The largest CH was found in family S. The minimum CW was observed in the male in family SA. In females, the CH of family A, family SA and family AS were not significantly different (*P* > 0.05). In males, the CH in family A and family AS was not significantly different (*P* > 0.05).

**Table 2:** Carapace length, carapace width, and carapace height of *P. monodon* (mean ± SD)

Family	Carapace length (cm)		Carapace width (cm)		Carapace height (cm)	
	Male	Female	Male	Female	Male	Female
FS	2.86 ± 0.5933^C^	2.94 ± 0.4352^b^	1.63 ± 0.3698^C^	1.67 ± 0.4655^b^	1.82 ± 0.3341^C^	1.87 ± 0.2435^b^
FA	2.63 ± 0.4356^B^	2.62 ± 0.3976^a^	1.39 ± 0.2967^A^	1.43 ± 0.3914^a^	1.67 ± 0.4586^B^	1.72 ± 0.3769^a^
FSA	2.40 ± 0.6210^A^	2.61 ± 0.4311^a^	1.33 ± 0.2655^A^	1.39 ± 0.3415^a^	1.49 ± 0.2912^A^	1.61 ± 0.3915^a^
FAS	2.65 ± 0.2346^B^	2.67 ± 0.5865^a^	1.49 ± 0.3113^B^	1.55 ± 0.2993^a,b^	1.69 ± 0.3649^B^	1.70 ± 0.4438^a^

A, B, and C were significantly difference among males, a, b, and c in females

**Fig. 1:**
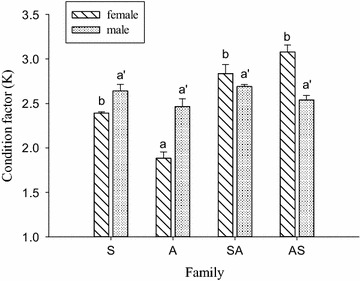
Condition factor (K) for male and female *P. monodon* (*S* family S, *A* family A, *SA* family SA, *AS* family AS)

The condition factor (K) was shown in Fig. 1. In females, the highest condition factor was observed in family AS (3.07 ± 0.07), and lowest condition factor was found in family A (*P* < 0.05). In males, the condition factor was not significantly different between all families (*P* > 0.05).

The exponent b values lie between 2.705 and 2.945 estimated in present study all well fall into the ranges in other penaeid species (Daud and Ang 1995; Primavera et al. 1998; Abohweyere and Williams 2008; Gopalakrishnan et al. 2014; Sun et al. 2015). The results indicate that all shrimps from four families showed negatively allometric growth. However, the regression parameters varied between families. The shrimps in these four families had more weight increment in relation to length increment than reported in other cases as indicated by the slope value *b* = 2.237 in *P. monodon* cultured-loose-shell affected and *b* = 2.485 in *P. monodon* caught from wild (Gopalakrishnan et al. 2014).

Sexual dimorphism in size (female > male) has been documented for several shrimp species such as *P. aztecus* (Parrack 1979), *P. indicus* (Devi 1986), *P. longistylus* (Dredge 1990), *P. vannamei* (Chow and Sandifer 1991), and *Metapenaeus endeavouri* (Buckworth 1992). Farmer (1986) found that the *b* values were greater in males of *P. semisulcatus*, *M. affinis* and *Parapeneopsis stylifera* than in females of these species from artisanal and industrial fisheries in Kuwait. In contrast, Roongratri (1993) found no sex-based size differences in LWRs in species such as *P. latisulcatus*, *P. merguiensis* and *P. semisulcatus* in Thailand. Furthermore, evidence indicates that high genetic correlations were obtained between abdominal segment length and body weight (Gitterle et al. 2005; He et al. 2011). In this study, the body weight of males from family S was bigger than females from same family. On the contrary, females have a greater regression slope than males in the family SA (2.946 vs 2.728) and in the family AS (2.868 vs 2.705) (Table 1). Although the b-values of females is bigger than males in family SA and AS, the CL, CW and CH were also greater than male. The big carapace is not conducive to effective growth or output. In the present study, the offspring of selected breeding families were cultured under controlled environment with same rearing protocol, the affection of abiotic parameter has been excluded. In this study, the alternation of the parameter in the LWR may be caused by the genetic inheriting and hybrid effects. This may suggest that the parameter in the LWR could be used in selective breeding, but may needs to be further investigated.

Previous research shows that females have higher condition factor values than males in grow-out cultured shrimp (Wang and Fang 1996). In the loose-shelled and wild *P. monodon*, the males have higher condition factor values than females (Gopalakrishnan et al. 2014). In the present study, the condition factor was not significantly different between males and females in same family. Furthermore, in comparison of condition factor between different families, the highest condition factor was observed in the females of family AS, and lowest condition factor was observed in the females of family A. This may cause by the hybrid effects between different geographic separated breeding families.

## Conclusions

The present study evaluated the LWRs and condition factor of *P. monodon* from four breeding families. The LWRs of *P. monodon* from four families followed a negative allometric trend. Base on analyzing the growth-related traits and condition factor in the current study family SA and family AS are suggested to be in good condition. This may be explained by superiority of intercross. The condition factor of family AS was higher than family SA in females, opposite in males, While neither was found to be significant. Even so, the present results may also be considered that hybrid effects existing in geographic separated breeding families.
